# Concurrent shifts in wintering distribution and phenology in migratory swans: Individual and generational effects

**DOI:** 10.1111/gcb.15151

**Published:** 2020-06-09

**Authors:** Rascha J. M. Nuijten, Kevin A. Wood, Trinus Haitjema, Eileen C. Rees, Bart A. Nolet

**Affiliations:** ^1^ Department of Animal Ecology Netherlands Institute of Ecology (NIOO‐KNAW) Wageningen The Netherlands; ^2^ Wildfowl & Wetlands Trust Slimbridge UK; ^3^ Postkast 104 Haapsalu 90501 Estonia; ^4^ Department of Theoretical and Computational Ecology Institute for Biodiversity and Ecosystem Dynamics University of Amsterdam Amsterdam The Netherlands

**Keywords:** *Cygnus columbianus bewickii*, generational shift, global warming, individual plasticity, short‐staying, short‐stopping

## Abstract

Range shifts and phenological change are two processes by which organisms respond to environmental warming. Understanding the mechanisms that drive these changes is key for optimal conservation and management. Here we study both processes in the migratory Bewick's swan (*Cygnus columbianus bewickii*) using different methods, analysing nearly 50 years of resighting data (1970–2017). In this period the wintering area of the Bewick's swans shifted eastwards (‘short‐stopping’) at a rate of ~13 km/year, thereby shortening individual migration distance on an average by 353 km. Concurrently, the time spent at the wintering grounds has reduced (‘short‐staying’) by ~38 days since 1989. We show that individuals are consistent in their migratory timing in winter, indicating that the frequency of individuals with different migratory schedules has changed over time (a generational shift). In contrast, for short‐stopping we found evidence for both individual plasticity (individuals decrease their migration distances over their lifetime) and generational shift. Additional analysis of swan resightings with temperature data showed that, throughout the winter, Bewick's swans frequent areas where air temperatures are c. 5.5°C. These areas have also shifted eastwards over time, hinting that climate warming is a contributing factor behind the observed changes in the swans' distribution. The occurrence of winter short‐stopping and short‐staying suggests that this species is to some extent able to adjust to climate warming, but benefits or repercussions at other times of the annual cycle need to be assessed. Furthermore, these phenomena could lead to changes in abundance in certain areas, with resulting monitoring and conservation implications. Understanding the processes and driving mechanisms behind population changes therefore is important for population management, both locally and across the species range.

## Graphical abstract

We demonstrate that Bewick's swans showed a significant and extensive eastward shift of 12.9 km/year in their wintering range in NW Europe over the past ~50 years. Simultaneously, individuals have shortened their stay in the wintering range by almost 9 weeks through arriving later and departing earlier in recent years.

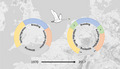

## 1 INTRODUCTION

Rapid global environmental change is affecting ecosystems, communities and species worldwide (Walther et al., 2002). Changes in temperature are of particular importance because of their influence on species' distribution (Chen, Hill, Ohlemuller, Roy, & Thomas, 2011; Hickling, Roy, Hill, Fox, & Thomas, 2006; Maclean et al., 2008; Parmesan & Yohe, 2003), abundance (Pounds, 2001; WWF, 2018), phenology (Visser & Both, 2005) and extinction risk (Thomas et al., 2004).

For migratory species that visit geographically discrete areas during their annual cycle, differing rates of change and the unpredictability of favourable conditions among these areas can create severe challenges (Robinson et al., 2009). If rates of change are not correlated between the areas these animals frequent, it is impossible for them to predict the time and place with optimal conditions (Kölzsch et al., 2015). This can lead to a mismatch with optimal circumstances (i.e. peak food abundance) because the cues they use change at a different rate (i.e. temperature at staging sites; Both, Bijlsma, & Visser, 2005), or do not change at all (i.e. day length; Post & Forchhammer, 2008). It has been found that migratory species that are declining tend to also be those that are showing weak or no phenological changes (Møller, Rubolini, & Lehikoinen, 2008), especially when migratory diversity within a species is small (Gilroy, Gill, Butchart, Jones, & Franco, 2016).

Changes in the distribution and/or phenology of a population can be the result of individual plasticity in the use of space and time. In the absence of individual plasticity, generational change must be the mechanism driving shifts in space or time (Gill, Alves, & Gunnarsson, 2019). Generational change can arise from recruits consistently using different schedules than the previous generations, or by differential mortality. Both change the frequency of individuals with a certain state (e.g. distribution or timing), leading to an observed population change in this property (Gill et al., 2019).

Range shifts have been documented for many taxa (Chen et al., 2011; Parmesan & Yohe, 2003). One specific form of a range shift in migratory species is ‘short‐stopping’: *A range shift that involves shortening of the migratory route, and is qualified by season (winter or breeding) and degree (full or partial)* (Elmberg, Hessel, Fox, & Dalby, 2014). Partial winter short‐stopping has been suggested to be driven by climate warming, and has been documented in a variety of ways in waterfowl (Fox et al., 2016; Lehikoinen et al., 2013; Pavón‐Jordán et al., 2019; Podhrázský et al., 2017). Fitness benefits may arise from shorter migration distances, as the annual cycle becomes less time‐constrained (Tomotani et al., 2018) and individuals can make better predictions of environmental conditions in geographic areas further along their route (Visser, Perdeck, van Balen, & Both, 2009). However, unforeseen harsh conditions in the areas closer to the breeding grounds could also be a risk and lead to high mortality (Suter & van Eerden, 1992).

In addition to changing distributions, migratory species can also show shifts in phenology in response to changing conditions (Diehl, 2019; Jonzén et al., 2006). Phenological changes can be studied in all aspects of the annual cycle (Tomotani et al., 2018) such as breeding (Dunn & Winkler, 2010) and the onset of migration (Cotton, 2003; Tombre et al., 2008). When phenological changes concern departure or arrival in a certain area (e.g. breeding or wintering), this can lead to a longer or shorter duration of time spent in an area. For the latter, we will use the term ‘short‐staying’ throughout this paper, in line with the term short‐stopping as defined by Elmberg et al. (2014), comprising *A phenological change in arrival or departure (or both) that involves shortening of the duration of time spent in a certain area*. Arrival or departure have been studied mainly with regard to the breeding area of birds (e.g. Jonzén et al., 2006; Xu & Si, 2019), but changes in the wintering area are relevant too (Stirnemann, O'Halloran, Ridgway, & Donnelly, 2012), as most migratory species are bound to a tight annual cycle and could thus benefit from shortening the time spent in their winter quarters if warming would permit them to do so (Norris & Taylor, 2005).

Apart from assessing that changes are taking place, it is important to identify the drivers and mechanisms behind such changes. In this context ‘drivers’ refer to the environmental steering factors such as temperature (Lehikoinen et al., 2013; Visser et al., 2009) or precipitation (Jónsson & Afton, 2015), acting either directly or indirectly (for example via food abundance) on where and when individuals are present. Alternatively, ‘mechanism’ refers to individual plasticity or generational shifts which are behind the changes observed at the population level. Individual plasticity can cause population level changes when the drivers to which individuals respond change consistently in a specific direction (Gienapp, Teplitsky, Alho, Mills, & Merilä, 2008). Generational shifts can cause changes in the spatial or temporal distribution of a population when the frequency of individuals with a certain spatial or temporal state changes, such as when environmental conditions alter the frequency of recruits with differing states, or the mortality rates associated with these states (Gill et al., 2019). Both may change the frequency of individuals with a certain state (e.g. distribution or timing).

For instance, individual black‐tailed godwits (*Limosa limosa islandica*) are consistent in their migration timing over their lifetime but the proportion of juveniles recruiting into two stopover sites changed, leading to a population pattern of advanced spring arrival (Gill et al., 2013). In another population of black‐tailed godwits (*Limosa limosa limosa*), juveniles started using a stopover site proportionally more than adults and continued using these sites into adulthood, leading to an observed distribution shift at the population level over time (Verhoeven et al., 2018). Gill et al. (2019) concluded that the observed changes in distribution and phenology of migratory species likely result from generational shifts, as opposed to individual plasticity, as individual fidelity in the use of space and time is thought to be strong (Winger, Auteri, Pegan, & Weeks, 2019). However, we expect that generational shifts are less prevalent in species that migrate in families where young individuals follow their parents and thus ‘copy’ their routes and schedules (‘states’) in early life. Shifts in range and phenology could arise in such a system through individual plasticity at any life stage, recruits changing state once independent of their parents, or through changes in mortality across individual states.

The Bewick's swan (*Cygnus columbianus bewickii*) is one of the largest migratory bird species using an energy‐intensive flapping flight mode, which requires stopovers for refuelling during migration, resulting in a tight annual cycle (Hedenstrom & Alerstam, 1998; Nuijten et al., 2014). A major decrease in numbers for the population which winters in NW Europe and breeds on the European Russian tundra, resulted in the development of an International Single Species Action Plan (Nagy et al., 2012) which identified threats to the population through expert assessment and called for research to determine the cause of the decline. Studies to date indicate that neither annual survival nor reproduction seem to have been solely responsible for the decline (Wood, Newth, Hilton, Nolet, & Rees, 2016; Wood et al., 2018), which has shifted the attention to the wintering grounds. Given the strong family bonds and site fidelity in this species (Rees, 2006), it was long thought that there would be relatively little flexibility in the use of wintering sites over the lifetime of individuals and their offspring. Recent drastic declines in numbers at some traditional wintering areas, and an absence of conclusive reasons for these trends, gave greater weight to the hypothesis that the swans were short‐stopping in their wintering range (Augst, Hälterlein, & Fabricius, 2019; Wahl & Degen, 2009; Wood et al., 2019). International census data revealed that Bewick's swans wintered further north but not east in milder winters, but, although numbers significantly decreased at the western edge and increased at the eastern edge, did not result in conclusive evidence for a north‐easterly shift in winter distribution over time, perhaps because counts were conducted only once every 5 years and hence the study had limited statistical power with which to detect such shifts (Beekman et al., 2019). In this study, we use ring‐resightings as a more powerful data source with hundreds of sightings every year, to analyse whether concurrent occurrences of winter short‐stopping and short‐staying are evident in this avian migrant, based on the hypotheses that the swans currently arrive later, depart earlier and stay further east (i.e. closer to the breeding grounds) in winter than before. Importantly, the ring‐resightings also enable us to analyse whether individual plasticity or generational shifts provide the mechanism for changes in the birds' phenological patterns. In addition we test the hypothesis that climate warming is the environmental driver for the observed changes by determining the mean air temperature where individual Bewick's swans occur, and comparing the shifts in this temperature with their range and phenology shifts.

## 2 MATERIALS AND METHODS

### 2.1 Ringing schemes

We used two long‐term monitoring programmes for Bewick's swans, one for leg‐rings and one for neck‐bands. In both schemes individuals were aged at capture as either juvenile, yearling or adult based on the presence or absence of grey plumage respectively (Rees, 2006). The leg‐ring programme, which commenced in 1970, was led by the Wildfowl & Wetlands Trust (WWT) in the United Kingdom. Rings were fitted both at wintering sites in the United Kingdom and on the breeding grounds in northern Russia. A total of 3,998 individuals are present in this data set, which were resighted 36,933 times outside the WWT's wetland reserves. The sightings within WWT reserves were explicitly excluded for this study, due to a different detection method (bill pattern rather than neck‐ or legring ID). The neck‐band ringing programme was initiated by TH (since 1989) and continued by NIOO‐KNAW (since 2005), and neck‐bands were applied in the Netherlands and Germany. In total, 843 individuals fitted with neck‐bands were resighted 38,148 times. A network of professional and amateur ornithologists across the winter range in northwest Europe resighted the marked birds and the scheme organizers collated reports of these resightings into a database. The resighting numbers in this study represent unique resightings per swan per day; if a swan was registered more than once a day (for example by multiple observers) only one sighting was included in the analyses. The resighting probability of tagged individuals differs between the two ringing schemes because the larger neck‐bands generally are easier to read in the field than leg‐rings: leg‐rings have a mean (±95% CI) resighting probability of 0.70 (±0.02) and neck‐bands 0.91 (±0.01), with no apparent trends over time (Wood et al., 2018). In this study we combined data from all individuals of both ringing schemes. Since we were interested in the overall patterns in the presence of the swans in time and space, the difference in resighting probability between swans marked with leg‐rings versus those with neck‐bands was not likely to influence the results. Where differences in the outcomes could occur, we analysed resightings of leg‐rings and neck‐bands separately, to determine whether the results were consistent irrespective of the marking method.

### 2.2 Short‐stopping

To assess the occurrence and extent of short‐stopping in this migratory species, we used three different methods. For the first two methods we selected resightings only for the months December and January in each winter season, to prevent short‐staying from influencing the results. In this subset, 3,865 individuals with a total of 26,366 resightings were present (leg‐ring: *N* = 3,216/15,364; neck‐band: *N* = 649/11,002 for individuals and resightings respectively). For the third method, all resightings in the winter half‐year (October–March) were included.

In the first method (M1), we calculated the mean average geographic location of the resightings per winter season, and tested whether longitude and/or latitude showed a directional change towards the north and/or east over time (see Fox et al., 2016) with the following model:

(1a) $${\text{Lat}}_{i}∼\beta_{0}\;+\;\beta_{1}Y_{i}\;+\;\varepsilon_{0i},$$

(1b) $${\text{Lon}}_{i}∼\beta_{0}\;+\;\beta_{1}Y_{i}\;+\;\varepsilon_{0i},$$

where Lat and Lon represents latitude (for assessing a northward shift) and longitude (for assessing an eastward shift) respectively, β_0_ represents the intercept, β_1_ is the slope for the dependency and *Y* is the winter season, and where subscript *i* refers to the winter season of the measurement and ε_0_ *_ij_* is the residual error term.

For the second method (M2), we created a grid with cells of 0.5° × 0.5° (lower left corner 10.5°W, 50.5°N; upper right corner 11.5°E, 58.5°N; WGS84; Supplementary Material S3) and counted the proportion of resightings per year in each gridcell for every winter season during the study period. Only winter seasons in which >5 gridcells contained resightings were included in the analysis (in total 44 winter seasons), to allow for an evaluation of the overall pattern in the winter area. We then calculated the trend in the proportion of observations within each gridcell that had resightings in >5 seasons (to be able to detect such a trend). The slope of this trend for each of the gridcells, weighted by the SE of the slope, was tested against longitude to see whether gridcells towards the east had more positive slopes (indicating an increase in the proportion of resightings in those locations) than gridcells in the west with the model

(2) $$S∼\beta_{0}\;+\;\beta_{1}\text{Lon}\;+\;\varepsilon_{0},$$

where *S* represents the slope within a gridcell, and Lon represents the longitude of that gridcell. Outlier Slimbridge was excluded from the analysis (see Section 4).

Thirdly (M3), we calculated the distance from each wintering location (i.e. each resighting) to the breeding grounds (see Podhrázský et al., 2017), as this corresponds directly to the definition of short‐stopping (Elmberg et al., 2014). We used a fixed location in the breeding area (Narjan‐Mar: 53.1°N, 67.7°E) and calculated the great‐circle (orthodromic) distance between each resighting recorded during the winter half‐year (Oct–Mar) and this point. The distances were limited to <4,000 km (i.e. the western boundary of Ireland) and >1,700 km (i.e. the distance to a known stopover site in Estonia). We selected the maximum distance for each individual for each winter season and analysed both the within‐ and between individual variation in these distances over time (Equation 3; *N* = 3,839 individuals, 14,302 resightings)

(3) $${\text{MD}}_{\mathrm{ij}}∼\beta_{0}\;+\;\beta_{W}\left(Y_{\mathrm{ij}}-Y_{j}\right)\;+\;\beta_{B}Y_{j}\;+\;u_{0j}\;+\;\varepsilon_{0ij},$$

where MD is the maximum migration distance per individual per winter season *Y*, β_0_ is the intercept, β_W_ is the estimate for within‐individual changes, *Y_ij_* is measurement in year *i* from individual *j*, *Y_j_* is the average year of measurement for individual *j*, β_B_ is the estimate for between‐individual changes and *u*_0_ *_j_* is the random intercept (van de Pol & Wright, 2009).

### 2.3 Short‐staying

To test whether Bewick's swans changed their winter phenology by shortening their duration of stay in the wintering grounds, we also used the resighting database described earlier, including both leg‐ringed and neck‐banded birds. To reduce the underestimation of wintering duration based on resightings (since the resighting rate is never a 100%) we only included individuals that were recorded >9 times in a winter season (Oct–Mar). We used this selection (43% of all resightings) to ensure that only birds for which the resighting data provided an overview of their presence in a particular winter season were included in the analysis, thus excluding birds with occasional resightings or with observations concentrated on a few days only. We defined the wintering range, and thus the area that the birds arrived at or departed from, as the area west of 12°E because this boundary included almost all winter resightings (see Figure 2) whilst being distantiated from the first well‐known migratory stopover sites (i.e. in the Baltic states). The first resighting of an individual to the west of this boundary was labelled as ‘arrival’, and the last as ‘departure’, for that particular swan and winter season. All departures in November/December (*N* = 29) and all arrivals in January/February (*N* = 188) were removed from the resulting data set, on the basis that they were attributable to (a lack of) individual observations, rather than to the timing of the birds' movements. In total, 672 individuals and 1,634 unique swan‐season combinations were included in this analysis. We analysed both the within‐ and between‐individual variation in arrival, departure and winter duration over time (Equation 4; *N* = 672 individuals, 1,634 observations)

(4) $$D_{\mathrm{ij}}∼\beta_{0}\;+\;\beta_{W}\left(Y_{\mathrm{ij}}-Y_{j}\right)\;+\;\beta_{B}Y_{j}\;+\;u_{0j}\;+\;\varepsilon_{0ij},$$

where *D* is the response variable (either arrival, departure or duration) per individual per winter season, β_0_ is the intercept, β_W_ is the estimate for within‐individual changes, *Y_ij_* is measurement *i* from individual *j*, *Y_j_* is the average year of measurement for individual *j* and β_B_ is the estimate for between‐individual changes (van de Pol & Wright, 2009).

### 2.4 Mechanisms behind short‐stopping and short‐staying

To study potential mechanisms behind short‐stopping and short‐staying, we compared the average population change in migration distance or winter duration respectively, with the rate of change in these variables in individuals over their lifetime within the study (see Gill et al., 2019). We used the statistical models provided by van de Pol and Wright (2009) to study within‐ and between individual variation for migration distance and winter duration (Equations 3 and 4). In addition, we applied the following models:

(5) $${\text{MD}}_{\mathrm{ij}}∼\beta_{0}\;+\;\beta_{W}Y_{\mathrm{ij}}\;+\;\left(\beta_{B}-\beta_{W}\right)Y_{j}\;+\;u_{0j}\;+\;\varepsilon_{0ij},$$

(6) $$D_{\mathrm{ij}}∼\beta_{0}\;+\;\beta_{W}Y_{\mathrm{ij}}\;+\;\left(\beta_{B}-\beta_{W}\right)Y_{j}\;+\;u_{0j}\;+\;\varepsilon_{0ij},$$

in which the (β_B_ − β_W_) term now assesses whether β_B_ and β_W_ are significantly different from each other (van de Pol & Wright, 2009). We interpreted a significant result for β_W_ as support for individual plasticity in the trait. In the absence of individual plasticity, generational shifts have to be causing a population shift.

When evidence for either of the mechanisms was found, we applied the same models (Equations 3 and 5 for migration distance; Equations 4 and 6 for winter duration) to the age classes (age determined at capture) separately to see whether there were particular groups of individuals driving the observed changes in either migration distance or winter duration. Lastly, we also analysed data of individuals with data in both their year of capture and the year thereafter because we hypothesized that if individual plasticity plays a role, particularly the yearlings would be prone to show this since adult Bewick's swan are believed to be site‐faithful and juveniles migrate with their parents and consequently follow their migratory schedule (Rees, 2006).

### 2.5 Temperature as an environmental driver

To study our hypothesis that climate warming drives the observed population changes in the Bewick's swan population we looked at air temperature as a proxy. For this, we matched all resightings west of 12°E in each winter season to the mean daily temperature recorded at the specific time and place of the resighting, using the R package RNCEP (Kemp, Emiel van Loon, Shamoun‐Baranes, & Bouten, 2012). Additionally, we collected temperature data for the whole of the wintering range and modelled the position of the isotherms over time to see if changes therein would match a shift in the swans' distribution. For the isotherm modelling we used the E_OBS v18.0e data set with 0.1° × 0.1° resolution grids with daily mean temperatures (Cornes, van der Schrier, van den Besselaar, & Jones, 2018), and averaged all daily maps for each winter (December and January) over the study period (1970–2017) and predicted the temperatures based on a regression model:

(7) $$T_{i}∼\beta_{0}\;+\;\beta_{1}{\text{Lat}}_{i}\;+\;\beta_{2}{\text{Lon}}_{i}\;+\;\beta_{3}{\text{Lat}}_{i}^{2}\;+\;\beta_{4}{\text{Lon}}_{i}^{2}\;+\;\beta_{5}Y\;+\;\beta_{6}{\text{Lon}}_{i}*Y\;+\;\varepsilon_{0},$$

where *T* is the mean average winter temperature in °C, Lat and Lon represent the latitude and longitude of the 0.1° × 0.1° gridcell *i*, and *Y* is the winter season (1970–2017; 1970 representing the winter of 1970–1971).

## 3 RESULTS

### 3.1 Short‐stopping and underlying mechanism

**FIGURE 1:**
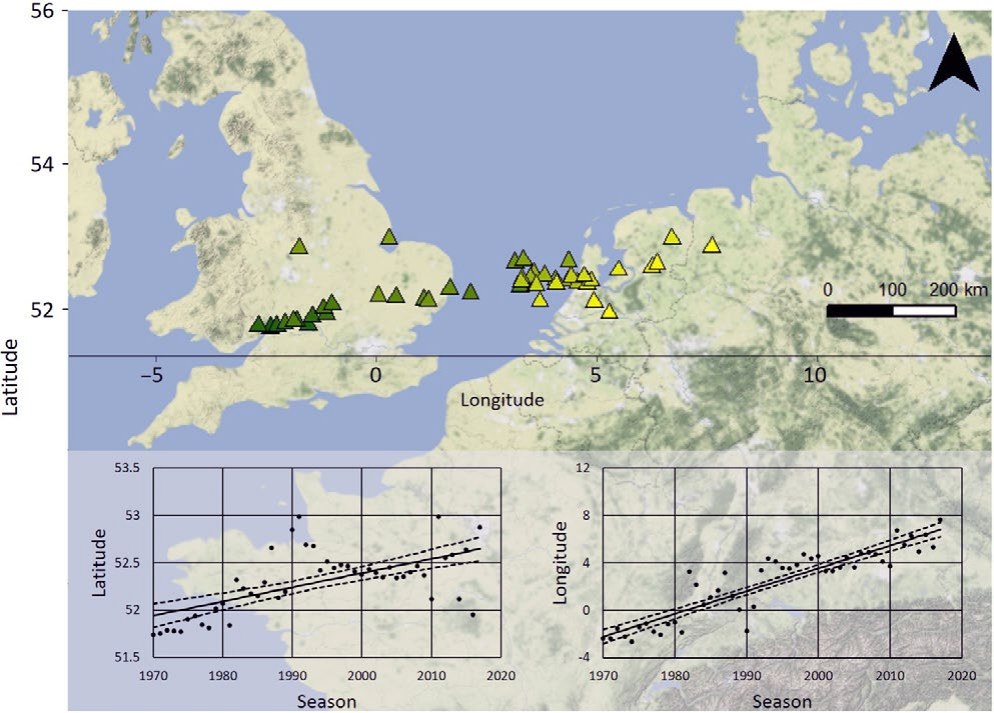
Mean winter location of midwinter (Dec–Jan) resightings in winter seasons 1970/71–2017/18 (M1 in main text). The colour gradient represents time, where dark green is the first winter (1970/71) and yellow is the last winter in the analysis (2017/18). Sample size (resightings per year) ranged between 48 and 1,539 (see Table S1.1 for an overview per season). The panel shows the latitudinal (left) and longitudinal (right) shift over time, with a regression (solid line) and 95% CI of the regression line (dashed lines). Both shifts are significant (see text)

Based on the mean resighting location for each winter (M1, Figure 1), the mean location of the wintering population of Bewick's swans in NW Europe shifted, both in latitude (*F*_1,46_ = 29.63, *p* « .001) and longitude (*F*_1,46_ = 218.8, *p* « .001; Table S4.1). Over the 48 years of the study, the slopes of the shifts in degrees latitude and longitude amounted to 0.015 ± 0.003 (mean ± *SE*) and 0.192 ± 0.013 (mean ± *SE*) respectively. Estimating the first and the last locations based on this regression and calculating the great circle distance between them equals a north‐eastward shift of 618 km, corresponding to a mean short‐stopping rate of 12.9 km/year. The sample sizes per winter season can be found in Supplementary Material S1; a similar analysis conducted for the two ringing schemes separately is presented in Supplementary Material S2. The latter analyses confirmed that swans in both ringing schemes show a similar eastward shift (longitude); however, the northward shift (latitude) was absent in the neck‐band data set. Including or excluding birds ringed in the breeding grounds rather than the wintering grounds did not change the results (Supplementary Material S2), thus they were included.

**FIGURE 2:**
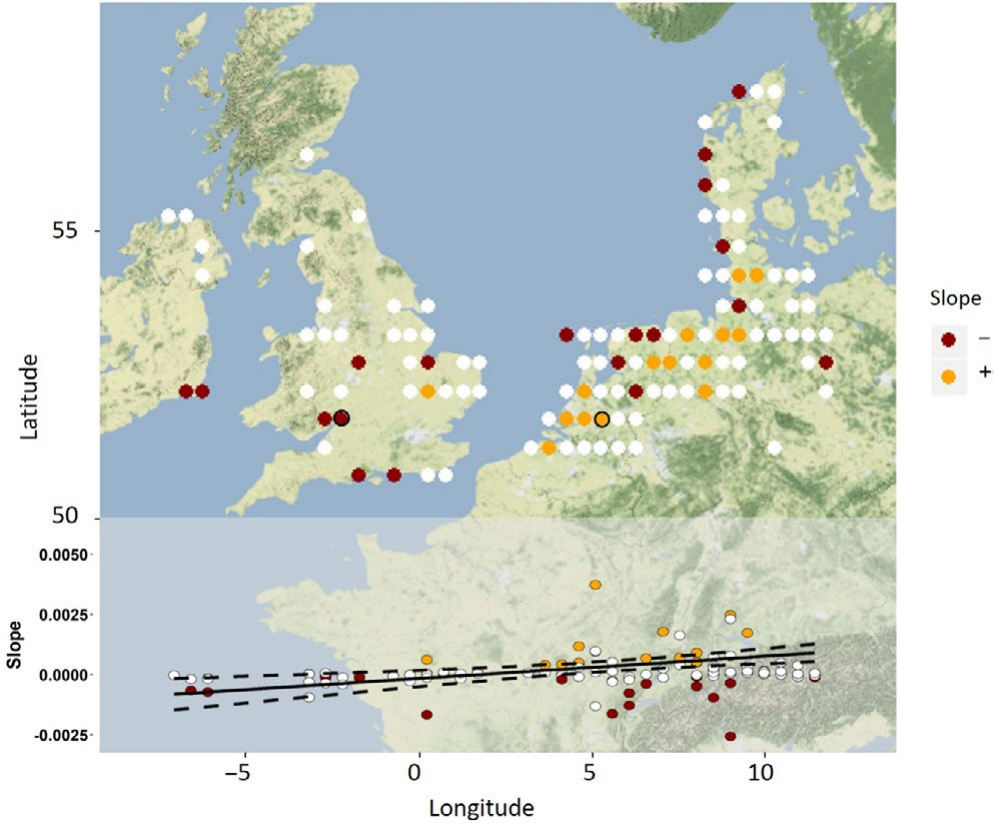
Trend of the proportion of resightings done per year in winter (Dec–Jan) in a theoretical grid over NW Europe (M2 in main text). Coloured dots represent gridcells with resightings in >5 seasons within the study period. The colour shows the trend of the slope of the proportion of resightings in that gridcell over time, with red being a significant (*p* < .05) negative slope (−) and orange a significant positive slope; white dots have non‐significant slopes. Slope values are visualized in the bottom panel of the figure, where the colours again indicate the significant trends. The solid black line in the bottom panel indicates a significant increase in the (weighted) slope value from west to east (see text). Dashed black lines represent the 95% CI of the regression. The red location with the black outline (main figure) is Slimbridge, a WWT site and the location with the largest decrease in proportion of resightings over time (slope −0.0166; excluded from bottom graph and analysis). The orange location with the black outline is in the province of Noord‐Brabant in the Netherlands, where the largest increase in the proportion of resightings took place (slope 0.0037)

Over the same time period, the proportion of resightings was more likely to increase over time in the eastern gridcells as compared to the western gridcells in the grid‐analysis (M2; *F*_1,114_ = 5.372, *p* = .022; Figure 2; Supplementary Material S4), confirming an eastward shift of the population between 1970 and 2017.

**FIGURE 3:**
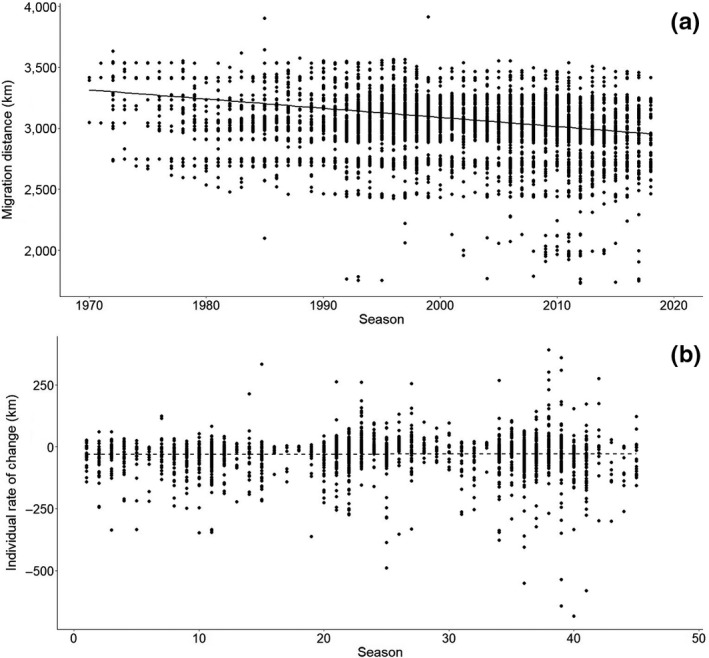
(a) Maximum migration distance of swans over time (M3). (b) Individual rate of change in maximum migration distance for individuals seen in >2 winter (Oct–Mar) seasons, each datapoint represents the slope of all observed migration distances for one individual over its lifetime in the study. Datapoints are weighted for number of seasons and plotted at the year of the first season (*x*‐axis). Detailed model output for both analyses is provided in Tables S4.3 and S4.4

Migration distances recorded for individual Bewick's swans decreased significantly over the study period (by −7.3 km/year; Figure 3a; see Table 1 for detailed model output), with mean distances of 3,324 km (estimate based on model output; Figure 3a) during the first season (1970/71), declining to 2,977 km in the last season of the study (2017/18). On average, based on this analysis (M3), the swans' migration is now 353 km shorter than in the early 1970s. The individual rate of change was significantly different from zero (−22 km/year, *p* < .001; Figure 3b) but did not change over the study period (*F*_1,2,050_ = 0.240, *p* < .001; Figure 3b; Table S4.4). When analysing the age classes separately for the difference in their migration distance between the year of their capture and the next year, both adults and yearlings decreased their migration distance significantly, juveniles did not (Supplementary Material S5).

**TABLE 1:** Results mixed models (Equations (3), (4), (5), (6)). (a) Model estimates for response variables individual migration distance, arrival day, departure day and winter duration with year as explanatory variable, either individually centred (β_B_) or based on the individual mean (β_W_). See Equations 3 and 4. (b) Model estimates for the same response variables but with year as explanatory variables and a slope estimate for β_B_ − β_W_, see Equations 5 and 6. As the model controls for all other effects in the model, year in this model is equal to the estimate for within‐individual variation (β_W_) in (a). The β_B_ − β_W_ effect in (b) now represents the difference between the within‐ and between individual variation (van de Pol & Wright, 2009)

Model	Parameter	Estimate	*SE*	*df*	*t*	*p*
(a)						
Mig. distance	Intercept	17,842.54	410.884	4,156.0	43.4	**<.001**
	β_W_	−14.83	0.626	11,237.1	−23.7	**<.001**
	β_B_	−7.37	0.206	4,148.4	−35.8	**<.001**
Arrival	Intercept	287.56	2.642	545.5	108.8	**<.001**
	β_W_	−0.09	0.241	1,099.8	−0.4	.721
	β_B_	1.11	0.077	607.4	14.4	**<.001**
Departure	Intercept	74.74	2.735	583.5	27.3	**<.001**
	β_W_	0.28	0.241	1,109.6	1.1	.252
	β_B_	−0.36	0.080	643.9	−4.6	**<.001**
Winter duration	Intercept	152.11	3.599	593.8	42.3	**<.001**
	β_W_	0.36	0.318	1,119.8	1.1	.252
	β_B_	−1.47	0.105	654.7	−14.0	**<.001**
(b)						
Mig. distance	Intercept	17,842.50	410.884	4,156.0	43.4	**<.001**
	Year	−14.83	0.626	11,237.1	−23.7	**<.001**
	β_B_ − β_W_	7.46	0.659	12,981.6	11.3	**<.001**
Arrival	Intercept	287.56	2.642	545.5	108.8	**<.001**
	Year	−0.09	0.241	1,099.8	−0.4	.721
	β_B_ − β_W_	1.20	0.253	1,295.1	4.7	**<.001**
Departure	Intercept	74.74	2.735	583.5	27.3	**<.001**
	Year	0.28	0.241	1,109.6	1.1	.252
	β_W_ − β_B_	−0.64	0.254	1,309.1	−2.5	**.012**
Winter duration	Intercept	152.11	3.599	593.8	42.3	**<.001**
	Year	0.36	0.318	1,119.8	1.1	.252
	β_B_ − β_W_	−1.83	0.335	1,316.4	−5.5	**<.001**

Bold values indicate significant results.

### 3.2 Short‐staying and underlying mechanism

**FIGURE 4:**
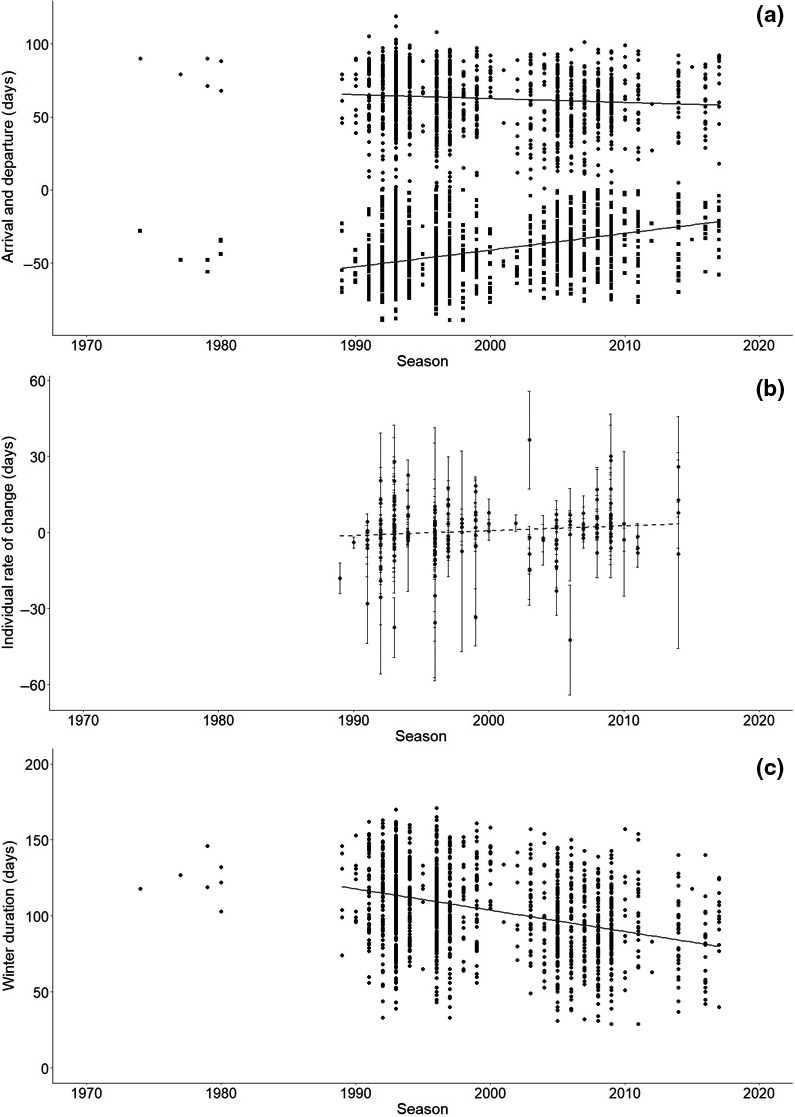
(a) Timing of arrival (bottom of figure a; <0) and departure (top of figure a; >0) for each winter season (*x* axis) for individually marked Bewicks swans resighted in the wintering area (west of 12°E). Days (*y*‐axis) are indicated as Julian days of the year counting the number of days from the first of January (1), with negative numbers indicating arrival days in December and November of the previous year. Solid lines show a significant later arrival (*N* = 1,634, *p* « .001) and earlier departure (*N* = 1,634, *p* « .001) over time. (b) Individual rate of change in winter duration for individuals seen in >2 winter seasons. Each datapoint represents the slope (±SE) of recorded winter durations over the lifetime of individual swans and is plotted on the first year this individual was seen (x‐axis). Individuals did not change their winter duration over their lifetime (see main text and Table S4.6 for detailed model output). (c) Winter duration of Bewick's swans over time (in number of days spent west of 12°E). Winter duration significantly decreased over the study period from 118.2 to 79.8 days (see text and Tables S4.4 and S4.5 for detailed model output)

The timing of arrival and departure in the wintering area changed markedly over the study period. During the years in which there were sufficient resightings for individuals seen outside WWT wetland reserves, the mean individual arrival date in the wintering grounds became progressively later (by 1.1 day/year; *p* < .001; Figure 4a; Table 1a), while departure on spring migration advanced (by 0.36 day/year; *p* < .001; Figure 4a; Table 1a). Overall, the duration of stay on the wintering grounds for the whole population was significantly reduced by 38.4 days since the winter of 1989–1990 (*p* < .001; Figure 4b; Table 1a). However, individuals did not change their winter duration over their lifetime (*F*_1,212_ = 2.403, *p* = .123; Figure 4c; Table S4.6).

### 3.3 Temperature as an environmental driver

**FIGURE 5:**
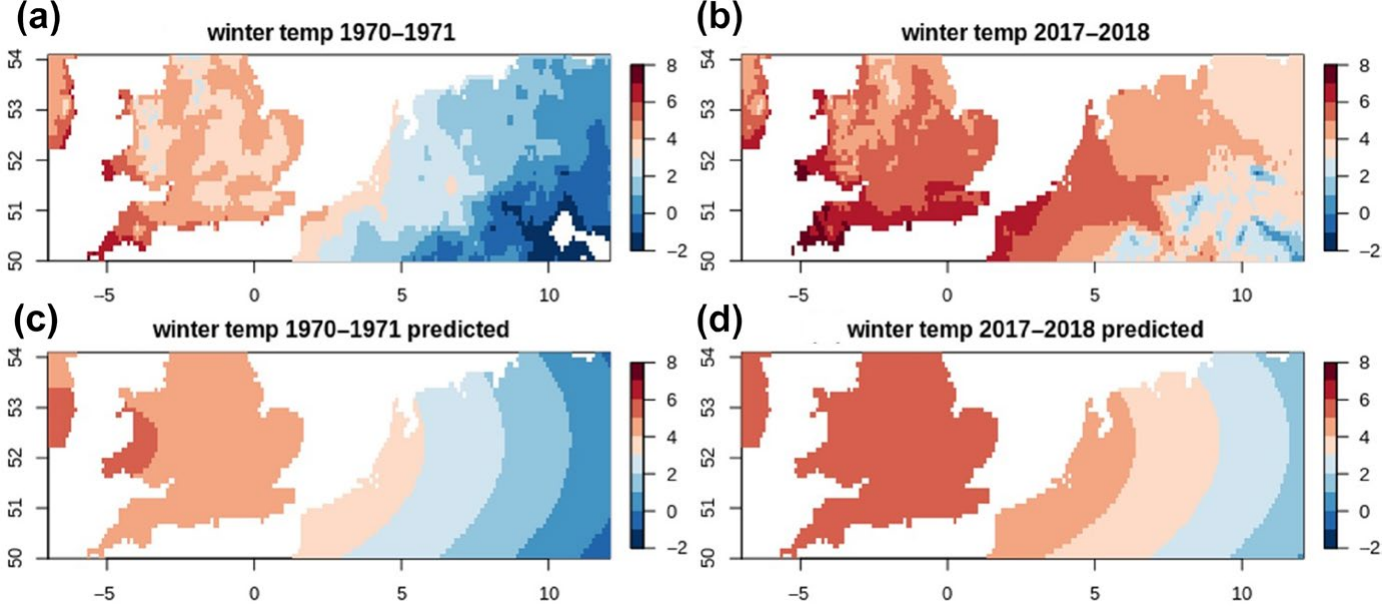
Actual (a, b) and modelled (c, d) isotherms in the first (1970/71; a, c) and the last (2017/18; b, d) winter season in our analysis. A comparison of (c) and (d) shows that the isotherms have shifted eastward

The temperatures at the resighting locations over the study period were remarkably similar (Supplementary Material S7). Median temperatures did not show a trend over time (*F*_1,46_ = 1.557, *p* = .218; Supplementary Material S7), and the swans thus seem to be present at a temperature of c. 5.5°C over the whole study period. Both the actual and the modelled temperatures in NW Europe showed increases over time, with isotherms shifting eastwards (Figure 5). For the 5°C isotherm of the modelled temperatures, which is closest to the temperature where the swans stage, this shift comprised 429.5 km over our study period (48 years), corresponding to 9.1 km/year (based on a fixed latitude of 52°N; Supplementary Material S8).

## 4 DISCUSSION

Our results confirm the hypotheses that the Bewick's swan population now arrives later, departs earlier and stays further east in winter when compared to half a century ago, thus confirming both short‐staying and short‐stopping in winter. When both processes are taking place concurrently, as in the present study, it is difficult to study them in isolation because to measure the changes in one, the other has to be fixed. For instance, to analyse short‐stopping one needs to fix the timing of winter in order to determine whether individuals change their location within this season, but this makes it by definition impossible to detect short‐staying. On the other hand, for short‐staying, the location of the wintering area needs to be fixed to allow detection of arrival and departure dates within this area, rendering it impossible to detect partial short‐stopping. To identify both processes separately, we only included midwinter resightings (i.e. resightings in December and January) in the first two analyses of short‐stopping, to prevent a change in the timing of arrival and departure from having an effect on our assessment of the swans' location. For the analyses of short‐staying, we fixed the wintering area as ‘west of 12°E’ for the whole study period. This boundary included almost all winter resightings (Figure 2), while being distantiated from the first well‐known stopover area of the swans (in the Baltic States). Moving this boundary to either 10°E or 15°E had little effect on the results (data not shown).

### 4.1 Short‐stopping

We found a major (>600 km) eastward shift in the swans' distribution over 48 years, corresponding to ~13 km/year (Figure 1). Winter range shifts of a similar order of magnitude have been documented in waders (~110 km in 20 years; Maclean et al., 2008). The extent of the swans' relocation can be partly but not entirely attributed to the fact that the North Sea between the United Kingdom and the Netherlands is an unsuitable wintering habitat for the birds. Over the course of our study period, the majority of the population stopped crossing the North Sea, and thus the mean winter location shifted to the east. The slight northward (latitudinal) shift (Figure 1) is probably influenced by migration direction. Analysing data from the two ringing schemes separately did not change the main result: both the leg‐ringed (1970–2017) and neck‐banded birds (1989–2017) stayed further east in more recent years, although only leg‐ringed birds also shifted significantly northwards (Supplementary Material S2).

Significant decreases in the proportion of resightings at particular locations (Figure 2) match information reported in other studies. For example Slimbridge, in southwest England, showed the strongest decrease in this analysis, and numbers of Bewick's swans are also decreasing at other sites in Britain, such as Ouse Washes (Wood et al., 2019), and only very small numbers are still visiting Ireland (Worden, Cranswick, Crowe, McElwaine, & Rees, 2006). In the Netherlands there has been a decrease of the swans' use of northern areas such as the Lauwersmeer and parts of Friesland (Tijsen & Koffijberg, 2015) in recent years (corresponding to the two red points in the north of the Netherlands in Figure 2). This is in line with the view that swans staging in the northern parts of the Netherlands used to cross the North Sea to winter in the United Kingdom, whereas birds staging further south generally do not do so (Tijsen & Koffijberg, 2015). Changes in habitat use and availability may also play a role (Clausen, Madsen, Cottaar, Kuijken, & Verscheure, 2018), and an increase in maize‐feeding may explain the proportional increase in the province of Noord‐Brabant (Koffijberg & Tijsen, 2018), in the south of the Netherlands, where the largest increase in the proportion of resightings was recorded (Figure 2). The finding that an increasing proportion of the resightings took place in northwest Germany (particularly the states Lower Saxony and Schleswig‐Holstein) is in line with observations made by Wahl and Degen (2009), and more recently by Augst Hälterlein and Fabricius (2019), who reported that this region is increasingly being used as a wintering area by Bewick's swans.

In agreement with the definition of short‐stopping provided by Elmberg et al. (2014), we found a significant shortening of the population migration distance to the breeding grounds, of >350 km over the study period (Figure 3). This corresponds to 11%–13% of the total migration distance and could potentially reduce constraints on the swans' annual cycle, leaving them with more opportunity to respond to changing circumstances. It was notable to us that the largest maximum distances did not seem to change too much over the study period. We believe that this may be due to the historically fixed location of the long‐term monitoring scheme at WWT Slimbridge in the United Kingdom, so that swans that visit this place have a high chance of being recorded. In addition, swans that are caught and subsequently resighted at or near Slimbridge, which particularly in recent years is towards the western edge of the swans' wintering range, provide the maximum distance data included in the analysis. If the level of short‐stopping showed in this study continues, we are aware that our catch locations may not provide a representative subset of the population in future years.

The analyses of migration distances (Figure 3a) assume that the swans' breeding site did not change significantly over the study period. There is some evidence for migratory bird species that breeding ranges are shifting northwards (Hitch & Leberg, 2007; Lehikoinen & Virkkala, 2016). Additionally, bioclimate modelling predicts a major shift in potential breeding range of many European breeding birds towards the end of the 21st century (Huntley, Green, Collingham, & Willis, 2007). However, for Arctic breeding birds there is not much opportunity for a northward shift, due to the absence of landmass north of the current breeding areas (Huntley et al., 2007). Data on breeding distribution for the Bewick's swan is limited. Since resightings in the breeding area are rare, and surveys are only done postbreeding and irregularly (Mineyev, 1991), assessing such a shift of the breeding range is difficult, but first results of the European Breeding Bird Atlas do not indicate a shift (E. C. Rees and S. Rozenfeld, unpublished data). Results from tracking studies also do not suggest major changes (Nuijten et al., 2014; R. J. M. Nuijten et al., unpublished data).

We found evidence for both individual plasticity and generational change when assessing within‐ and between‐individual variation in migration distance over time. This means that individuals do change their migration distance over their lifetime, possibly in response to climate warming (Figure 5). It has always been thought that Bewick's swans are very site‐faithful (Rees, 2006), and further research is required to study whether the observed change in migration distance reflects individuals not using parts of their range that they have previously used (in the west), but remaining site‐faithful to the sites further east or whether they actually explore new areas. In addition to these individual effects, the frequency of individuals using sites in the west or east changed over time resulting in an overall observed population change (generational effect). Range shifts such as short‐stopping resulting from generational shifts can also be driven by environmental changes, for example when recruits respond differently to the circumstances than their predecessors or when the changes cause selective (dis)appearance of individuals. Both mechanisms (individual plasticity and generational shifts) have been found to co‐occur in barnacle geese as well where it was found that especially juvenile geese tended to switch to another staging area. Older geese were less likely to switch, but this probability did increase over time, suggesting individual plasticity (Tombre, Oudman, Shimmings, Griffin, & Prop, 2019). We found no clear differences between adults and yearlings, both age classes shortened their migration between the year of catch and the year after, suggesting that the between‐individual short‐stopping was driven by selective (dis)appearance. Whether selective disappearance (in the west) or selective appearance (in the east) takes place requires further research.

The three analyses of short‐stopping (Figures 1, 2, 3, 4, 5) show strong evidence for this process; however, the observed changes could potentially (at least partly) be due to changes in observer effort in time and space (Buckland, Magurran, Green, & Fewster, 2005). Unfortunately, we cannot control effectively for this confounding factor, but count data both from some key Bewick's swan wintering sites as mentioned above and from systematic 5‐year censuses show trends in numbers which correspond to our findings (Beekman et al., 2019). If changes in observer effort in time and space did occur, and it is indeed likely that they did, these trends support that this potential observer effect did not influence our analyses to the extent that incorrect conclusions were drawn. Interestingly, it was shown before that resighting probabilities for the different ringing schemes did not show a directional change over time (figure 4 in Wood et al., 2018), which supports our idea that changes in observer effort are of minor importance.

### 4.2 Short‐staying

In addition to shifting their winter distribution, we also found evidence that time spent in the wintering ground has shortened (Figure 4). Between 1989 and 2017, the time that the Bewick's swans spent west of 12°E has declined by an average of ~38 days. Extrapolation suggests that over the study period the swans shortened their wintering period by 2 months. As with short‐stopping, this could potentially make the annual cycle of these migrants less time constraint. Whether this is indeed the case, and whether potential benefits or repercussions at other times of the annual cycle do occur need further investigation.

Although (spring) arrival dates are frequently studied in migratory birds (Hüppop & Hüppop, 2003; Jonzén et al., 2006), less emphasis is put on the duration of stay in the non‐breeding range. Gordo and Sanz (2006) did, however, mention the importance of this measure of phenology and showed that two of the five migratory bird species included in their study changed the duration of stay in Spain. When different phases of the annual cycle shift with differing rates, this can lead to overlapping or diverting phases (Tomotani et al., 2018). Concerning the Bewick's swans in this study, we found a shortening of the wintering phase due to a later arrival in autumn and earlier departure in spring. Although autumn arrival changed by a larger extent, spring departure is thought to have more consequences for fitness as it could lead to more flexibility in timing during spring migration and enable early arrival on the breeding grounds, which could be beneficial in the light of current climate change given that early onset of breeding is associated with higher breeding propensity, increased clutch sizes and higher nesting success in waterbirds (Nolet, Schreven, Boom, & Lameris, 2020). However, environmental changes and potential population responses during spring migration and breeding season should be studied to substantiate this potential effect (Norris & Taylor, 2005).

In two populations of black‐tailed godwits it was found that, although individual birds did not change their phenology and site use over the years, new recruits in the population did (Gill et al., 2013; Verhoeven et al., 2018), suggesting generational shifts to be a mechanism behind the population change (Gill et al., 2019). Similarly, we did not find evidence for individual plasticity for short‐staying, suggesting that a generational shift is steering the observed changes on the population level (Figure 4). The observed changes are of the same magnitude as (spring departure) or higher than (autumn arrival) changes in spring arrival dates in a range of bird species that were considered to be within the range of potential microevolutionary change, but these bird species were much smaller and will have shorter generation times than Bewick's swans (Charmantier & Gienapp, 2014; Gienapp, Leimu, & Merilä, 2007). In order to assess whether microevolutionary change can account for such rapid changes information on heritability of the trait (i.e. pedigree information) and fitness differences (if any) between phenotypes need to be known (Gienapp et al., 2008; Merilä & Hendry, 2014). Since we do not have this information available for this population, we focused on individual behaviour here and assessing microevolutionary change is beyond the scope of this study.

### 4.3 Potential drivers

The 5°C isotherm, which is close to the air temperature where Bewick's swans were resighted, shifted in the same direction and with the same magnitude as the swans' wintering location. Shifts in this order of magnitude have been confirmed before for isotherms in Europe (Beniston, 2014). This suggests that the swans' short‐stopping and perhaps also short‐staying are driven by a change in temperature. If so, it could be a direct effect through, for example, decreased costs of thermoregulation in areas with higher temperatures, enabling the swans to winter further northeast than before. Although Bewick's swans are relatively large birds, with wide thermoneutral zones, studies in other swan species suggest that these birds have thermoregulation costs below 5°C (Bech, 1980; Nespolo, Artacho, Verdugo, & Castañeda, 2008). Alternatively, the correspondence with temperature could represent an indirect effect such as via food availability, as frozen water and fields during periods of <0°C temperatures may prevent swans from feeding on aquatic and terrestrial plants respectively. An earlier study including Bewick's swans based on count data did not find a distributional shift nor a relationship with milder winters (Pavón‐Jordán et al., 2019), but this may have been influenced by a grouping of both species and weather data in the analysis.

While changes in habitat availability are also known to be important for the distribution of waterfowl (Clausen et al., 2018; Fox et al., 2005), long‐term habitat data covering NW Europe were not available for studying this at the continental level. Future research could focus on the influence of habitat availability on within‐winter movements of Bewick's swans, to gain further insights into this factor (Nolet, Bevan, Klaassen, Langevoord, & Van Der Heijden, 2002).

## 5 CONCLUSION

The results of this study provide strong evidence for short‐stopping and short‐staying by Bewick's swans wintering in northwest Europe, and these spatial and temporal changes may have important consequences for our understanding of the dynamics of this population. In Bewick's swans winter area use (short‐stopping) was changing by individual plasticity and generational shifts, whereas arrival and departure in the winter area (short‐staying) was changing by generational shifts. This led to changes in abundance in both time and space (Augst et al., 2019; Beekman et al., 2019). Understanding the processes behind the rapid changes in abundance is therefore important for population monitoring, management and conservation.

## Supporting information

Supplementary Material

## Data Availability

The data that support the findings of this study are openly available on Dryad ‘Concurrent shifts in wintering distribution and phenology in migratory swans’, reference number https://doi.org/10.5061/dryad.dfn2z34xp (Nuijten, Wood, Rees, & Nolet, 2020).
