# Supplementary material for: Concurrent shifts in wintering distribution and phenology in migratory swans: Individual and generational effects
Source: Glob Chang Biol. 2020 Jun 9;26(8):4263–75. doi: 10.1111/gcb.15151 (PMC7384179; doi:10.1111/gcb.15151)
Supplement: Supplementary file 1 — Supplementary Material [file GCB-26-4263-s001.pdf]

Supplementary material S.1

Table S1.1 Number of resightings (N) short-stopping analysis based on mean location (latitude and longitude) of resightings per winter per season (method 1 in main text).

	Season	Seasonnr	Mean.lat	Mean.lon	N
1	19701971	1	51.74055	-2.40512	156
2	19711972	2	51.75693	-2.3889	61
3	19721973	3	51.78825	-1.53601	89
4	19731974	4	51.77742	-2.24422	165
5	19741975	5	51.77258	-2.67131	48
6	19751976	6	51.90814	-1.44842	76
7	19761977	7	51.93837	-1.12551	126
8	19771978	8	51.84574	-1.79373	247
9	19781979	9	51.81055	-2.06563	314
10	19791980	10	52.02035	-1.18665	184
11	19801981	11	52.07196	-0.99282	188
12	19811982	12	51.84266	-1.87047	117
13	19821983	13	52.32209	3.271671	146
14	19831984	14	52.22631	2.1483	337
15	19841985	15	52.17919	0.458765	281
16	19851986	16	52.14845	1.091358	218
17	19861987	17	52.29695	1.681535	286
18	19871988	18	52.65615	3.164477	107
19	19881989	19	52.13287	1.186956	203
20	19891990	20	52.19713	0.052245	237
21	19901991	21	52.85175	-1.74943	480
22	19911992	22	52.98735	0.296592	510
23	19921993	23	52.68782	3.358744	652
24	19931994	24	52.67473	4.383683	919
25	19941995	25	52.41917	4.093512	1225
26	19951996	26	52.51428	3.582494	718
27	19961997	27	52.44482	3.515402	703
28	19971998	28	52.47698	3.829205	1181
29	19981999	29	52.46947	4.746665	821
30	19992000	30	52.40592	4.393268	784
31	20002001	31	52.37406	4.570991	505
32	20012002	32	52.43313	3.32205	527
33	20022003	33	52.39657	3.302281	420
34	20032004	34	52.34543	3.638763	531
35	20042005	35	52.45495	4.447601	470
36	20052006	36	52.34317	3.644572	1128
37	20062007	37	52.35402	4.803036	1248
38	20072008	38	52.4026	4.896817	1503

39	20082009	39	52.46815	4.738608	1539
40	20092010	40	52.36552	4.097582	1082
41	20102011	41	52.11678	3.730362	1116
42	20112012	42	52.9878	6.72993	792
43	20122013	43	52.55287	5.514886	622
44	20132014	44	52.58836	6.272805	527
45	20142015	45	52.11357	4.959362	658
46	20152016	46	52.63933	6.397523	394
47	20162017	47	51.95124	5.30471	977
48	20172018	48	52.87718	7.639933	748

Supplementary material S.2

In the first method (method 1 in main text), we calculated the mean average geographic location of the Dec-Jan resightings per winter season, and tested whether longitude and/or latitude showed a directional change towards the north and/or east over time. To assess a potential effect of marker method (leg-ring or neck-band) or catch location (winter range or breeding range), we performed the analyses on these separate groups as well. First for the neck-bands only (Figure S2.1), second for the leg-ringed birds excluding those birds caught in Russia on the breeding grounds (Figure S2.2) and third for the leg-ringed birds caught in Russia (Figure S2.3). Results are presented in the text below the figures.

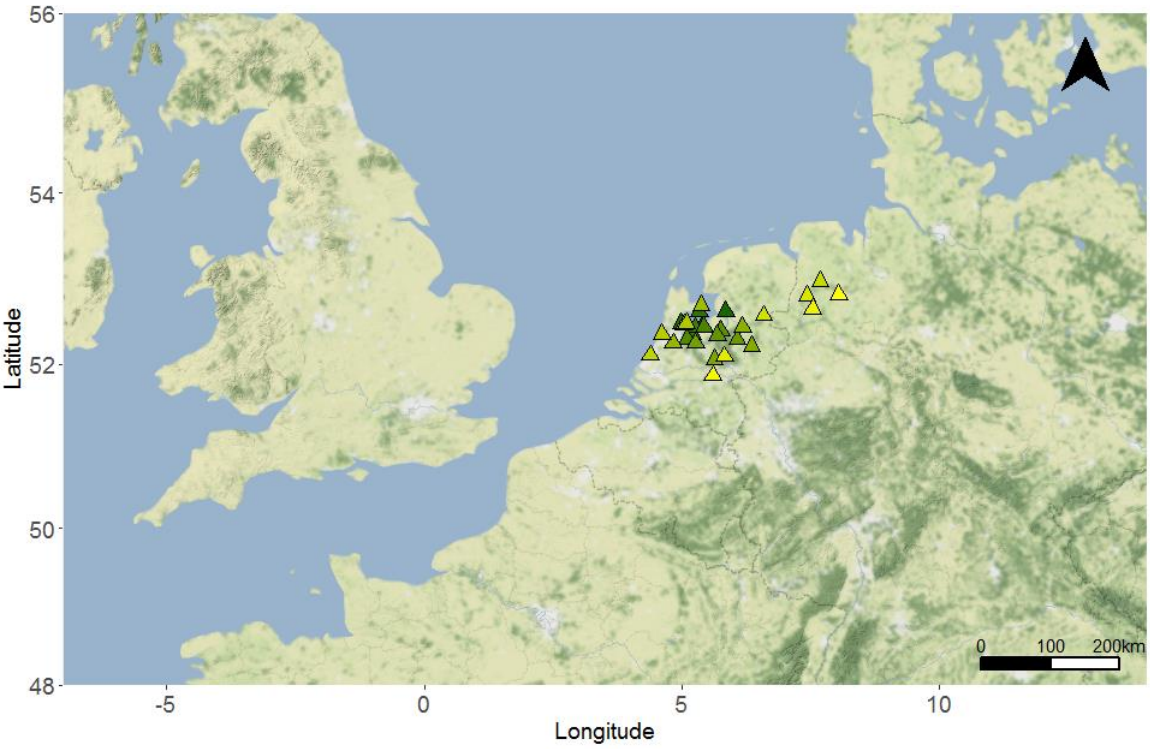

Figure S2.1. Mean winter location of swan resightings for neck-bands only (1989-2017). The colour gradient represents time where dark green is the first winter (1989/90) and yellow is the last winter in the analysis (2017/18). Longitudinal shift was significant ($F_{1\text{-}26}$ = 7.5, p = 0.011), latitudinal shift was not ($F_{1\text{-}26}$ = 0.0513, p = 0.823).

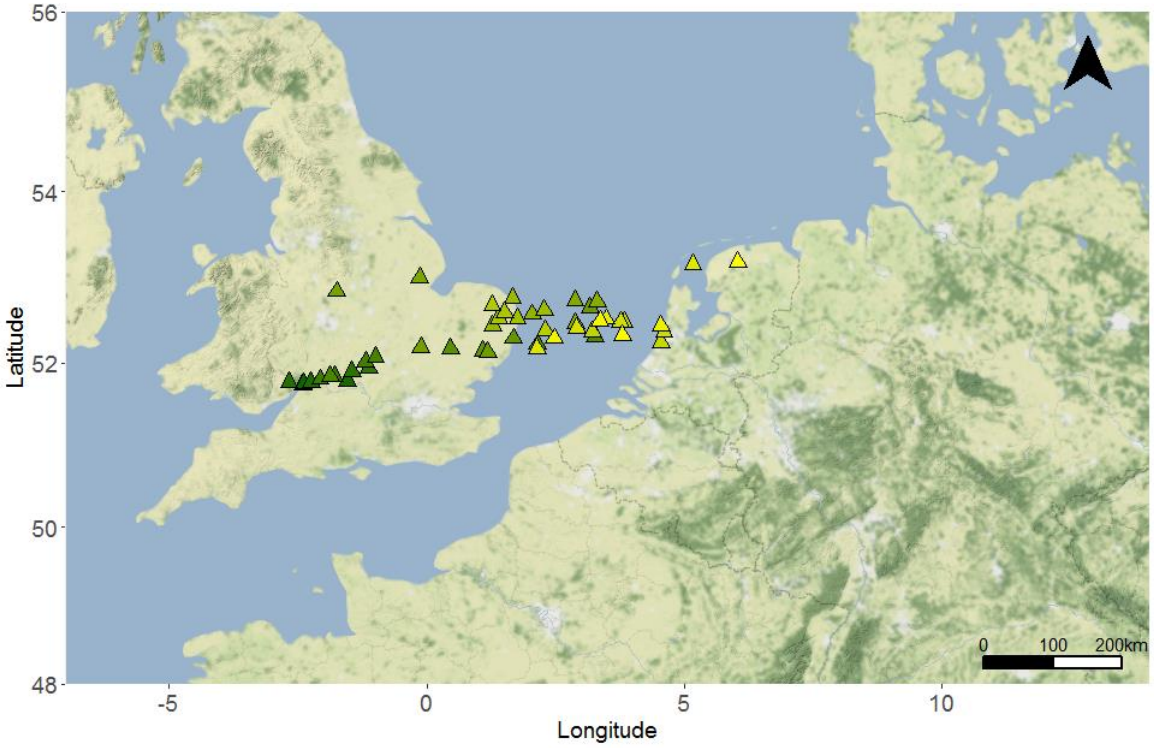

Figure S2.2. Mean winter location of swan resightings for leg-rings caught in wintering range (excluding catch sites in Russia; 1970-2017). The colour gradient represents time where dark green is the first winter (1970/71) and yellow is the last winter in the analysis (2017/18). Both latitudinal ($F_{1\text{-}46}$ = 31.22, p << 0.001) and longitudinal ($F_{1\text{-}46}$ = 219, p << 0.001) shifts were significant.

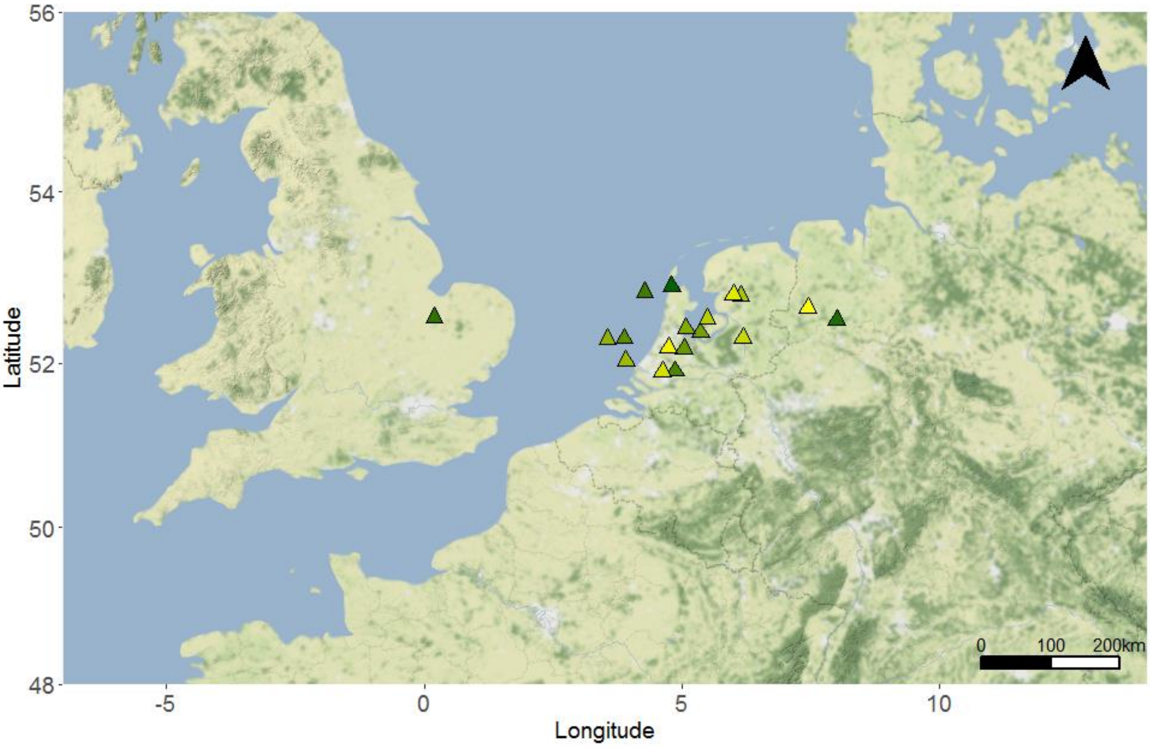

Figure S2.3. Mean winter location of swan resightings for leg-ringed birds ringed in Russia (1992-2017). The colour gradient represents time where dark green is the first winter (1992/93) and yellow is the last winter in the analysis (2017/18). Both latitude ($F_{1\text{-}16}$ = 0.4163, p = 0.528) and longitude ($F_{1\text{-}16}$ = 1.855, p = 0.19207) shifts were not significant. However, we noticed an odd pattern in the data, with two of the early years representing the furthest west and furthest east mean location. This might be caused by the small sample size for both number of resightings in December and January (299 individuals and 2098 resightings) and years (18 winter seasons in between 1992/93 and 2017/18) in this subset of the data. Especially in the early years (1992-2004) only five winter seasons were represented in the data, all of which had low numbers of resightings (max 19). However, if we exclude those years for the birds ringed in Russia the shifts are still not significant (latitude p = 0.44; longitude p = 0.0543), which might be caused by the short timescale (13 seasons; 2005-2017). Despite this specific group of leg-ringed birds showing no distribution shift, in- or excluding these in the main analysis did not change the overall results as presented in the main text.

Supplementary material S.3

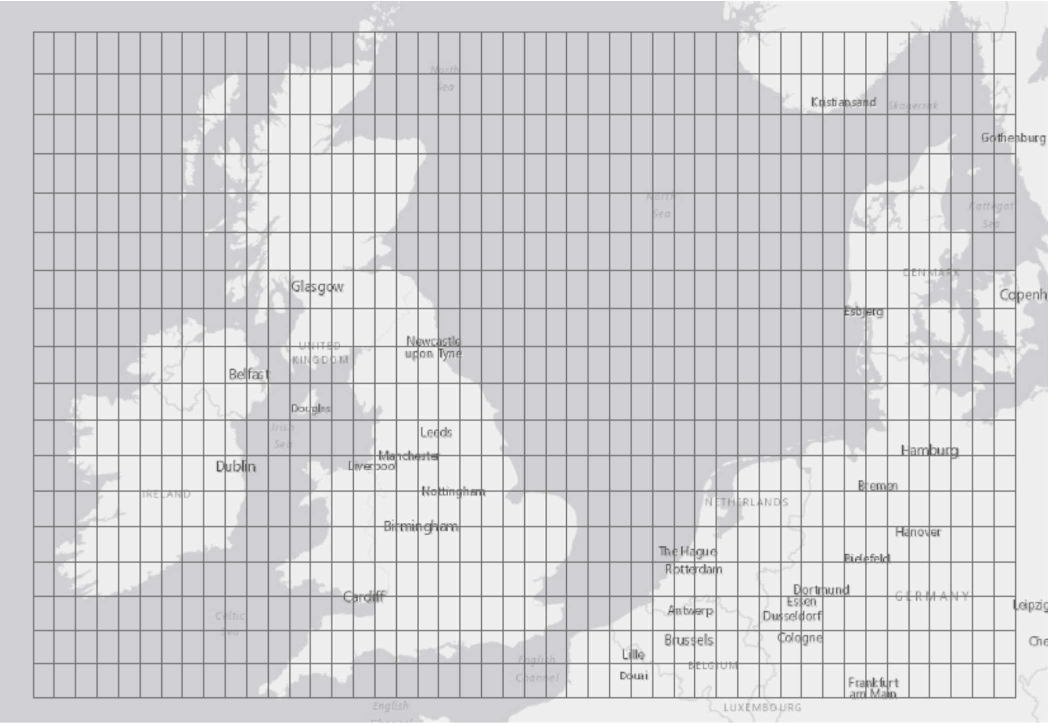

Figure S3.1 Geographical grid used in the second analyses for short-stopping. Cells are 0.5˚ x 0.5˚. The lower left corner has coordinates -10.5˚W, 50.5˚N, the upper right corner has coordinates 11.5˚E, 58.5˚N.

Supplementary material S.4

Table S4.1. Model estimates for response variables Latitude and Longitude of the mean location of resightings explained by time (winter season, expressed as Year). Method 1 in main text (see eqn. 1a and 1b and Fig. 1).

Model	Parameter	Estimate	*SE*	t	p
**Latitude**	Intercept	51.93	0.078	670.0	<.001
	Year	0.01	0.003	5.4	<.001
**Longitude**	Intercept	-2.42	0.365	-6.6	<.001
	Year	0.19	0.013	14.8	<.001

Table S4.2. Model estimates for response variable slope with explanatory variable Longitude of the gridcells. Method 2 in main text (see eqn. 2 and Fig. 2).

Model	Parameter	Estimate	*SE*	t	p
**Slope**	Intercept	-0.0002	0.0002	-1.0	0.339
	Longitude	0.00009	0.00002	3.7	0.003

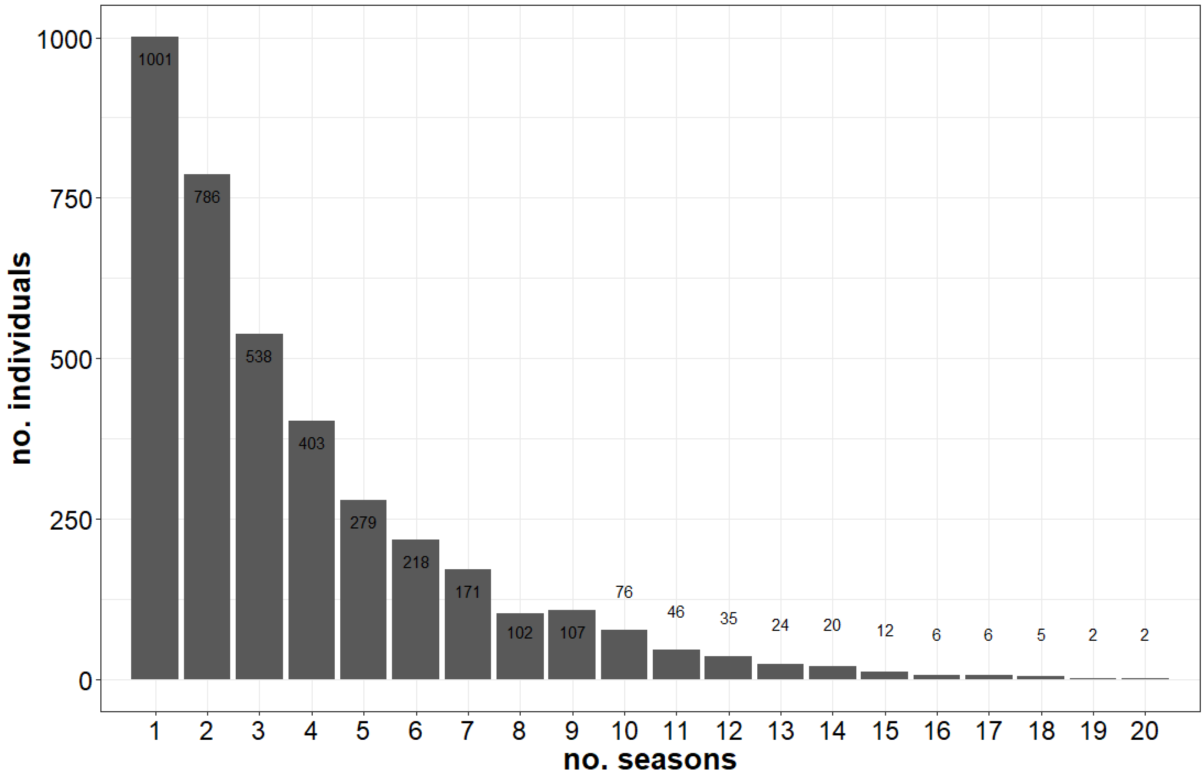

Figure S4.1. Number of years on which a migration distance for 3839 individually marked Bewick's swans has been recorded.

Table S4.3. Model estimates for response variable migration distance explained by time (winter season, expressed as Year). Method 3 in main text (see eqn. 3 and Fig. 3a).

Model	Parameter	Estimate	*SE*	t	p
**Mig. distance**	Intercept	18130.19	330.884	54.8	<.001
	Year	-7.5200	0.166	-45.4	<.001

Table S4.4. Model estimates for response variable individual migration distance explained by time (winter season, expressed as the first year in which a migration distance for an individual was recorded). Only individuals with a measurement in > 2 years were included for this analysis. Method 3 in main text (see eqn. 4 and Fig. 3b).

Model	Parameter	Estimate	*SE*	t	p
**Ind. mig. distance**	Intercept	-21.98	3.34	-6.6	<.001
	First year	-0.06	0.13	-0.5	0.625

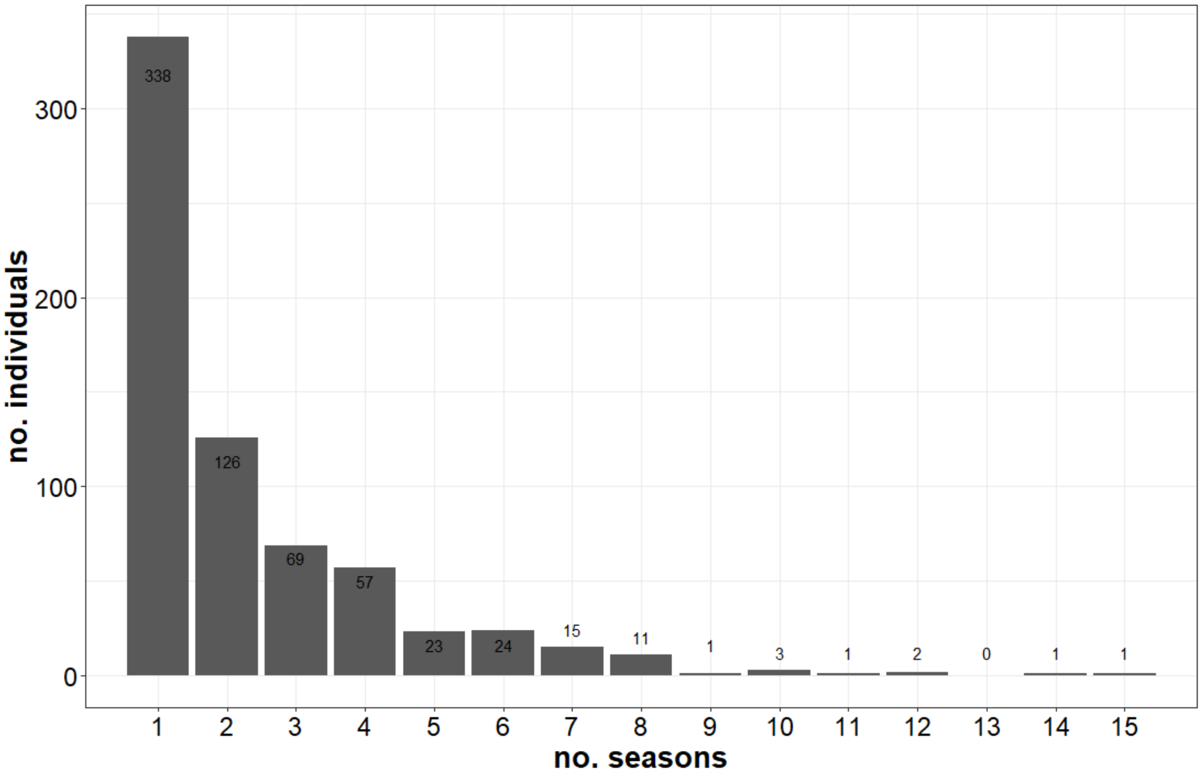

Figure S4.2. Number of years on which a winter duration 678 individually marked Bewick's swans has been recorded (1989-2017).

Table S4.5. Model estimates for response variable winter duration explained by time (winter season), expressed as the first year a winter duration was recorded for an individual. See eqn. 5 and Fig. 4 in main text.

Model	Parameter	Estimate	*SE*	t	p
**Winter duration**	Intercept	145.63	2.714	53.7	**<.001**
	First year	-1.37	0.087	-15.7	**<.001**

Table S4.6. Model estimates for response variable individual winter duration explained by time (winter season, expressed as the first year in which a winter duration for an individual was recorded). Only individuals with a measurement in >2 years were included for this analysis. See eqn. 4 and Fig. 4b in main text.

Model	**Parameter**	**Estimate**	***SE***	**t**	**p**
**Ind. Winter**	Intercept	-4.06	2.939	-1.4	0.169
**duration**	First year	0.15	0.099	1.6	0.123

Supplementary material S.5

Table S5.1. Model estimates for the difference in migration distance (ΔMD) between the year of catch and the year after for age classes Adult, Yearling and Juvenile over time. Age was determined at capture.

Table S5.1

Model	Parameter	Estimate	*SE*	t	p
ΔMD Adult	Intercept	-210.89	19.501	-10.8	<.001
	Year	2.61	0.654	4.0	<.001
ΔMD Yearling	Intercept	-318.79	57.003	-5.6	<.001
	Year	6.25	1.831	3.4	<.001
ΔMD Juvenile	Intercept	-177.73	33.251	-5.3	<.001
	Year	-0.08	1.257	-0.1	0.949

Supplementary material S.6

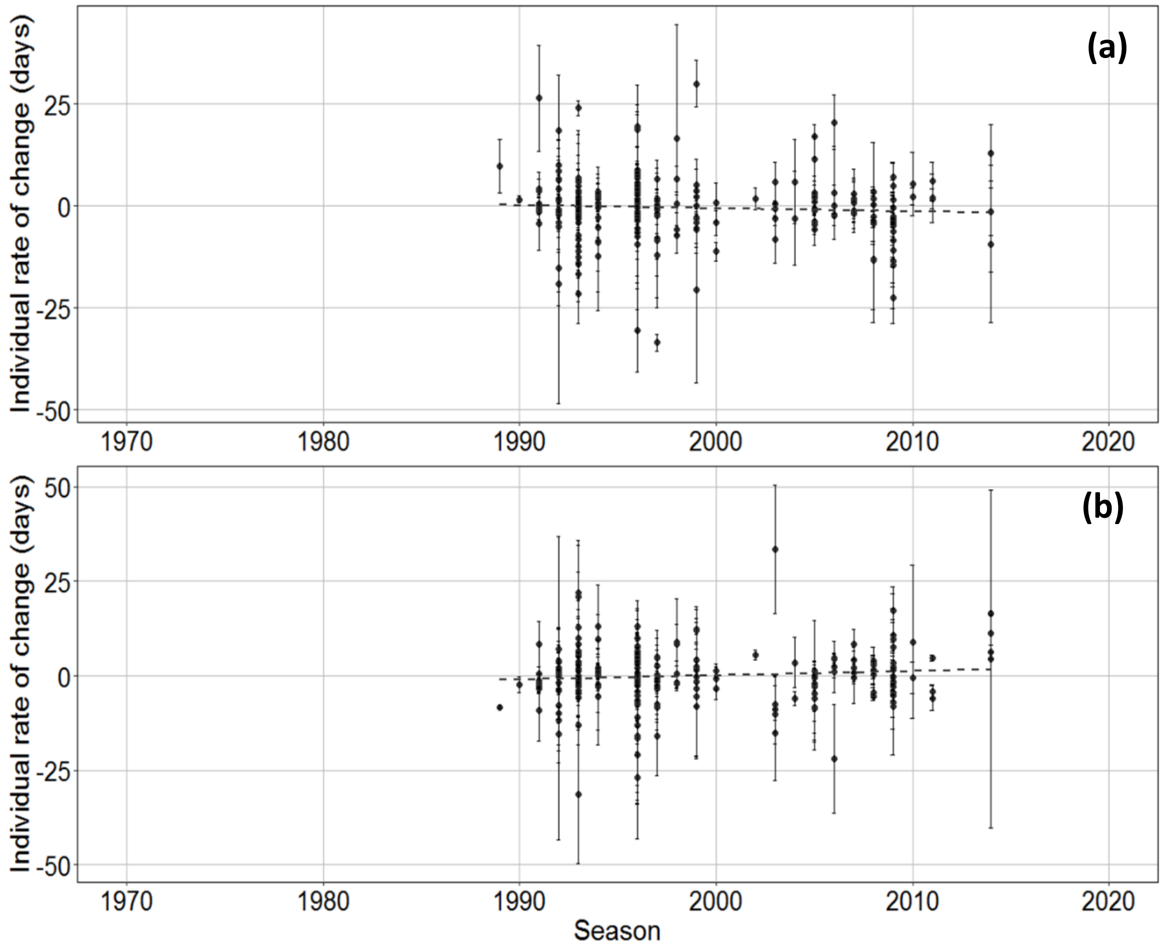

Figure S6.1 (a) Individual rate of change in arrival day, each datapoint represents the slope (±se) of arrival days for all seasons a specific individual was observed (data is weighted for number of seasons). Individuals did not change their arrival day over their lifetime (N = 214, p = 0.393). (b) Individual rate of change in departure day, each datapoint represents the slope (±se) of arrival day for all seasons a specific individual was observed (data is weighted for number of seasons). Individuals did not change their departure day over their lifetime (N = 214, p = 0.233).

Table S6.1. Model estimates for response variable individual arrival day explained by time (winter season, expressed as the first year in which a departure day for an individual was recorded). Only individuals with a measurement in >2 years were included for this analysis.

Table S6.1.

Model	Parameter	Estimate	*SE*	t	p
**Ind. arrival**	Intercept	1.61	2.235	0.7	0.471
	First year	-0.06	0.075	-0.9	0.393

Table S6.2. Model estimates for response variable individual departure day explained by time (winter season, expressed as the first year in which a departure day for an individual was recorded). Only individuals with a measurement in >2 years were included for this analysis.

Table S6.2.

Model	Parameter	Estimate	*SE*	t	p
**Ind. departure**	Intercept	-2.36	2.129	-1.1	0.272
	First year	0.086	0.072	1.2	0.233

Supplementary material S.7

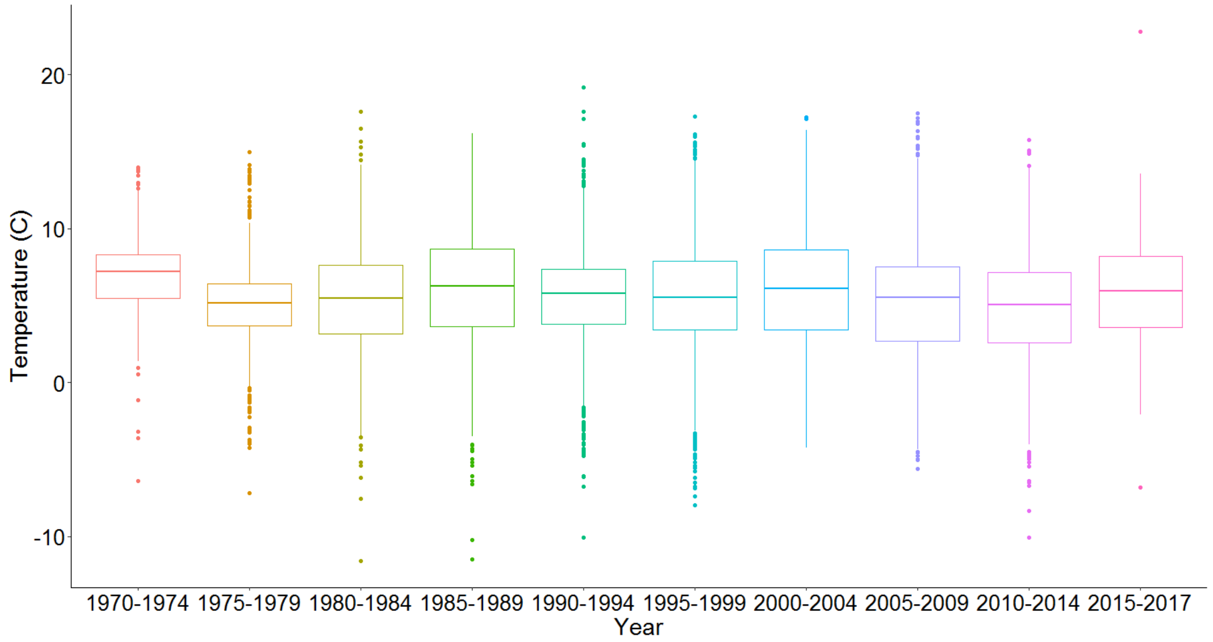

Figure S7.1. Temperatures at which individual Bewick's swans were resighted west of 12˚E, presented as boxplots aggregating 5-year periods. There was no significant change over time in the median temperature of the resightings over the study period ($F_{1,46} = 1.557$, $p = 0.218$; Table S6.1).

Table S7.1. Model estimates for response variable median winter temperature of resightings to the west of 12˚E with winter season (expressed as Year) as explanatory variable.

Table S7.1.

Model	Parameter	Estimate	*SE*	t	p
**Median temp.**	Intercept	38.09	26.071	1.5	0.151
	Year	-0.02	0.013	-1.2	0.218

Supplementary material S.8

Based on the actual temperatures in NW Europe in winter (averages for Dec-Jan in each winter from 1970/71 to 2017/18 inclusive) provided by the E_OBS v18.0e dataset (Cornes et al., 2018), we modelled the temperatures for all these winter seasons in order to reduce the effects of extreme years and obtain smoothed isotherms, for calculating the shift of these isotherms (Fig. 5 in main text).

To model the temperatures, we used the regression model:

$$T_i \sim \beta_0 + \beta_1 Lat_i + \beta_2 Lon_i + \beta_3 Lat_i^2 + \beta_4 Lon_i^2 + \beta_5 Y + \beta_6 Lon_i * Y + \varepsilon_0 \quad (1)$$

where T is the mean average winter temperature in °C, *Lat* and *Lon* represent the latitude and longitude of the 0.1˚ x 0.1˚ gridcell i, and Y is the winter season (1970-2017; 1970 representing the winter of 1970/71).

The model with the interaction between longitude and year (i.e. allowing longitudinal temperature changes to vary over the years) was significantly better than a model without this interaction ($p << 0.005$). All variables included in the model had a statistically significant effect on temperature (Table S8.1).

Table S8.1. Model estimates for response variable winter temperature of resightings to the west of 12˚E with winter season (expressed as Year) as explanatory variable.

Table S8.1.

Model	Parameter	Estimate	*SE*	t	p
**Winter temperature**	Intercept	-479.30	8.913	-53.8	**<.001**
	Latitude	17.06	0.342	49.9	**<.001**
	Longitude	-1.045	0.100	-10.5	**<.001**
	Latitude^2	-0.163	0.0033	-49.5	**<.001**
	Longitude^2	-0.013	0.0002	-81.6	**<.001**
	Year	0.019	0.0003	55.4	**<.001**
	Lon:Year	0.0004	0.00005	8.7	**<.001**
